# Distal Intersection Tenosynovitis: Surgical Insights From Five Cases

**DOI:** 10.3390/jcm14062110

**Published:** 2025-03-19

**Authors:** Julie Mercier, Agata Durdzinska Timoteo, Romain Baillot, Sébastien Durand

**Affiliations:** Department of Plastic and Hand Surgery, Centre Hospitalier Universitaire Vaudois, 1005 Lausanne, Switzerland; julie.mercier@chuv.ch (J.M.); agata.timoteo@chuv.ch (A.D.T.); romain.baillot@chuv.ch (R.B.)

**Keywords:** distal intersection tenosynovitis, extensor pollicis longus, extensor carpi radialis longus, extensor carpi radialis brevis, hand surgery, tenosynovectomy, magnetic resonance imaging

## Abstract

**Background:** Distal intersection tenosynovitis (DIT) is a rare and recently described condition that affects the extensor pollicis longus (EPL), extensor carpi radialis brevis (ECRB), and longus (ECRL). Based on surgical observations, this study aimed to provide new insights into its physiopathology. **Methods:** This was a retrospective study of all patients who underwent surgery for DIT at our institution from 2015 to 2024. Five patients were included in the study. **Results:** Wrist joint issues clearly explained the occurrence of DIT in three cases. Tendon lesions were observed either on the extensor carpi radialis brevis or extensor pollicis longus. **Conclusions**: These additional data complement the existing literature, which primarily focuses on the anatomical mechanisms of DIT without fully explaining its causes. Our observations suggest that wrist joint or bone disorders may play a significant role in its occurrence. Lesions in different tendons suggest the involvement of distinct pathological mechanisms.

## 1. Introduction

The extensor tendons of the fingers are located at the dorsal aspect of the wrist and separated into six distinct compartments. The second compartment contains the extensor carpi radialis longus and brevis tendons (ECRL and ECRB), which run between the first compartment and the Lister tubercle. The ECRL and ECRB tendons originate from the lateral epicondyle and insert, respectively, based on the second and third metacarpals. The third compartment is composed of the extensor pollicis longus tendon (EPL), which originates from the dorsal ulnar shaft and the interosseous membrane of the forearm and inserts into the distal phalanx of the thumb. The EPL tendon runs ulnar to the Lister tubercle, turning around it and changing direction toward the thumb to cross superficially over the tendons of the second compartment [1,2,3].

**Figure 1:**
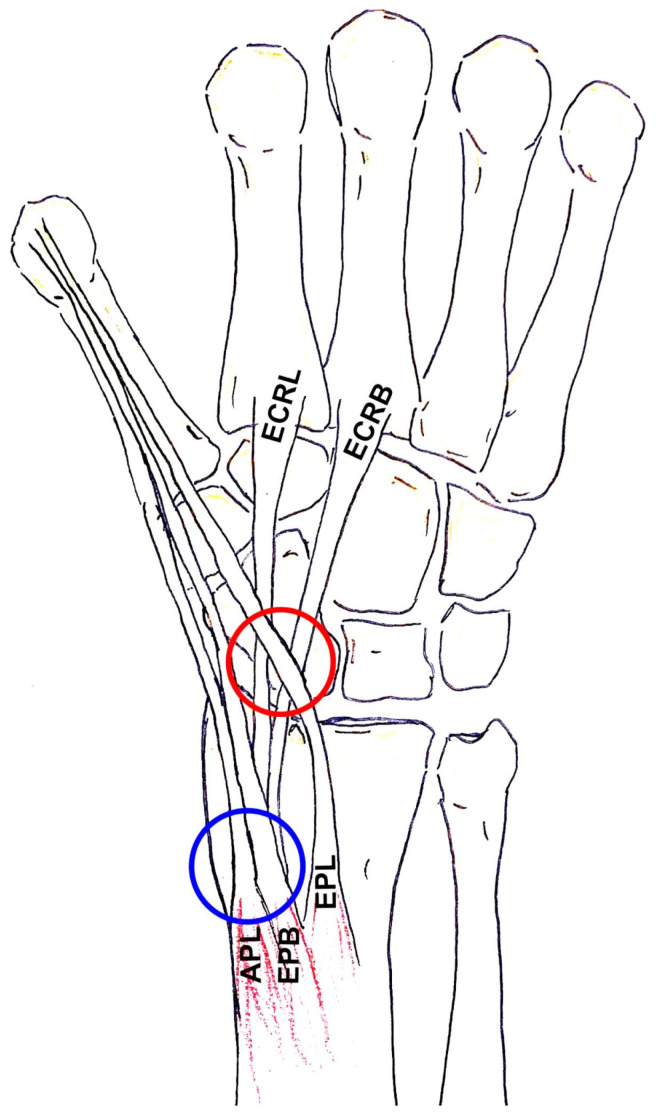
DIT (red circle) occurs between the tendon of the third extensor compartment (EPL) and the tendons of the second compartment (ECRB and ECRL). Intersection syndrome (blue circle) concerns the crossing of tendons of the first extensor compartment, the Abductor Pollicis Longus (APL), and the Extensor Pollicis Brevis (EPB) over the tendons of the second compartment (ECRB and ECRL).

In 2007, a group of radiologists from Philadelphia described distal intersection tenosynovitis (DIT) observed on the MRI of five patients [4]. Also called “distal intersection syndrome”, it refers to the concomitant tenosynovitis of the EPL, ECRL, and ECRB. This entity is distinct from intersection syndrome (Figure 1), initially described by Velpeau in 1842, which is situated on the dorsal aspect of the forearm, 4–8 cm proximal to Lister’s tubercule, and concerns the crossing of tendons of the first extensor compartment over the tendons of the second compartment [5]. According to recent anatomical studies, the strong correlation (*p* < 0.001) between the simultaneous presence of EPL tenosynovitis and ECRB/ECRL tenosynovitis [6] is likely related to the prevalence of the communicating foramen between the tendon sheaths of the ECRB and EPL [6].

DIT is a rare condition with an incidence of approximately 0.8% in wrist MRI examinations [6]. Clinical examination reveals dorsoradial pain of the wrist, tenderness over Lister’s tubercle, and eventually crepitus and local swelling or edema. It has also been identified as a potential predisposing factor for spontaneous EPL tendon rupture [7,8], although the precise pathophysiology remains unclear [7]. In this study, we report five cases of DIT and provide an intraoperative illustration of this condition. We discuss its pathophysiology based on case histories and surgical findings, highlighting the diagnostic and therapeutic implications of this under-recognized pathology.

## 2. Materials and Methods

The present investigation was a retrospective analysis of the institutional database of a single hospital. This manuscript was prepared following the STROBE guidelines. This study was conducted in accordance with the Convention of Helsinki. Ethical approval was not required by our Ethics Committee because human case series were reported (≤5). Informed consent was obtained from all subjects involved in the study. Written informed consent was obtained from the patients for the publication of this paper. A search of our institutional database identified five patients for this study who met our inclusion and exclusion criteria. The inclusion criteria were patients who presented with DIT confirmed by ultrasound and/or MRI and who had undergone surgery for DIT from December 2015 to December 2024. The exclusion criteria included patients with diffuse tenosynovitis affecting both the extensor tendons of the fingers and/or flexor tendons. No statistical analysis was performed, as this was merely a case series of five patients with a predominantly descriptive nature.

## 3. Results

**Case 1:**

**Figure 2:**
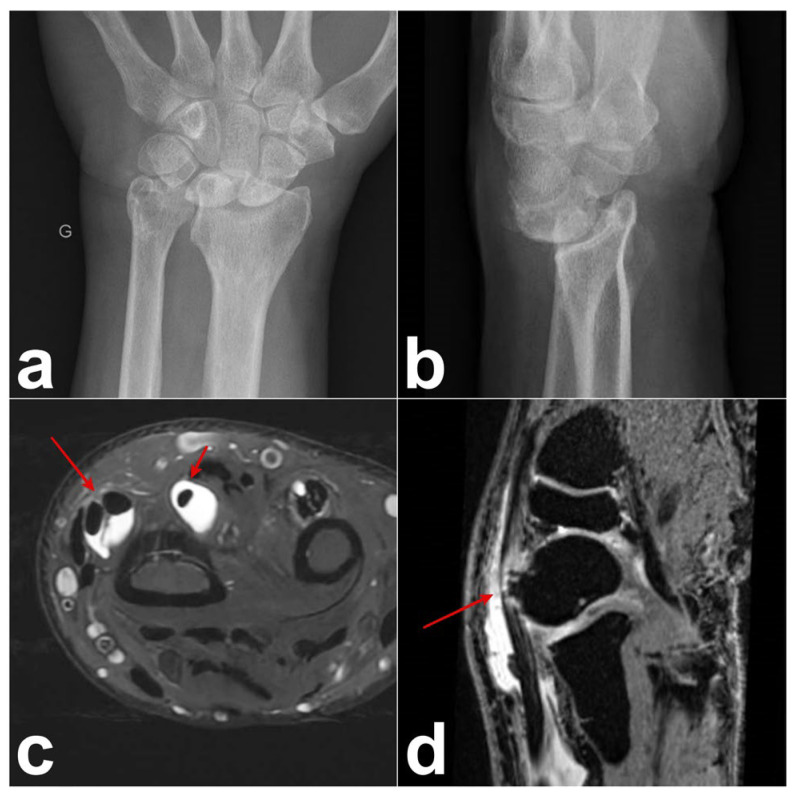
Case 1. (**a**,**b**) Preoperative radiograph showing malunion of the distal radius with dorsal subluxation of the wrist as well as radiocarpal and midcarpal arthritis. (**c**) Wrist MRI with axial T2-weighted fat-saturated gradient-echo sequence. DIT: Tenosynovitis of the EPL, ECRB, and ECRL (red arrows). (**d**) Wrist MR arthrogram (T1-weighted VIBE (Volumetric Interpolated Breath hold examination [VIBE) sequence with coronal orientation, isotropic resolution, and water excitation): dorsal subluxation of the scaphoid with signs of remodeling and its abrasive action on the ECRB tendon (red arrow).

**Figure 3:**
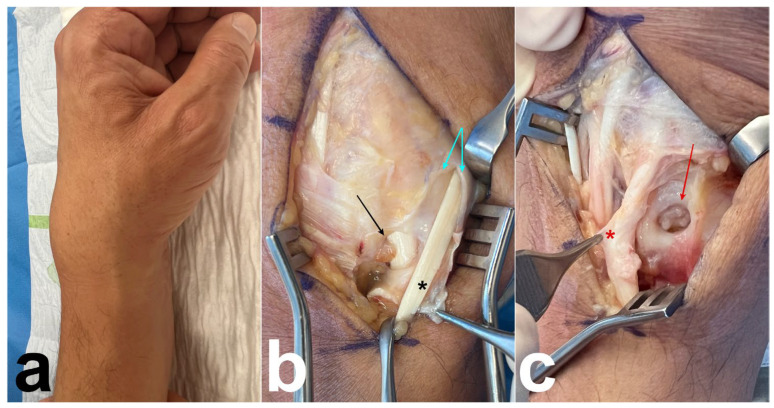
Case 1. (**a**) Preoperative photograph showing swelling of the dorsal aspect of the wrist. (**b**) Preoperative photograph; after opening the third compartment (blue arrows), an intact EPL tendon is observed (black asterisk). At the floor of the third compartment, the foramen communicating with the ECRB tendon is clearly visible (black arrow). Synovitis is present around ECRB. (**c**) Deeper, a perforation in the dorsal wrist capsule is observed (red arrow), revealing the remodeled scaphoid and damaged deep surface of the ECRB (red asterisk).

A 60-year-old male, a right-handed manual worker, visited our clinic with intense pain on the dorsal side of his left wrist after lifting heavy objects. Swelling of the dorsoradial aspect of the wrist and painful limited flexion and extension of the wrist were observed. Ultrasound revealed that the swelling was due to the presence of DIT. Radiographs and arthro-MRI highlighted the malunion of the distal radius following a prior fracture treated conservatively. This condition was associated with the dorsal subluxation of the wrist and radioulnar, radiocarpal, and midcarpal osteoarthritis (Figure 2). The proximal scaphoid exhibited structural changes and appeared aggressive toward the ECRB on MRI (Figure 2). Owing to the underlying bone issue, which could not be resolved with conservative treatment, we opted for a surgical procedure. A longitudinal dorsal incision was made and the extensor retinaculum was incised over the third compartment. The EPL was intact, and a communicating foramen was observed between the tendon sheaths of the ECRB and EPL (Figure 3). We observed a perforation at the level of the dorsal wrist capsule, creating direct contact between the proximal pole of the scaphoid and the deep surface of the ECRB (Figure 3). The deep aspect of the ECRB was severely damaged (Figure 3). Proximal row carpectomy, radio-capitate and radio-hamate arthrodesis using CCS screws and the partial denervation of the wrist by resecting the terminal branch of the posterior interosseous nerve were performed. Teno-synovectomy of the ECRB, ECRL, and EPL was conducted, and the subcutaneous transposition of the EPL was performed. Two months after surgery, arthrodesis was fused, the DIT disappeared, and the ultrasound findings normalized.

**Case 2:**

**Figure 4:**
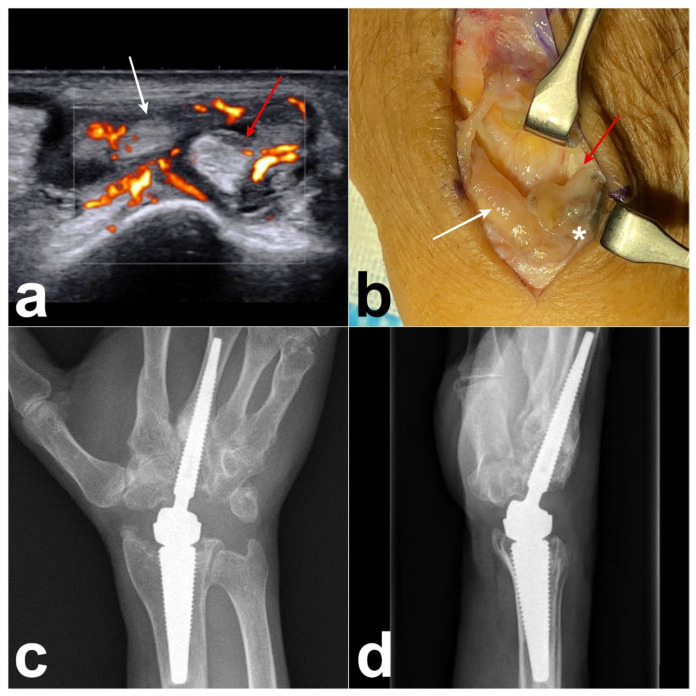
Case 2. (**a**) Ultrasonography reveals tenosynovitis around the EPL (white arrow), ECRB (red arrow), and ECRL. (**b**) Preoperative photograph showing the presence of tenosynovitis over the EPL (white arrow) and ECRB (red arrow), and associated with metallosis (white asterisk). (**c**,**d**) Postoperative radiograph showing the Motec wrist prosthesis with PEEK cup.

A 64-year-old right-handed man underwent a right wrist arthroplasty (Motec^®^ wrist joint prosthesis; Swemac, Linköping, Sweden) for severe radiocarpal and midcarpal osteoarthritis. One and a half years after the surgery, the patient presented with tenderness and edema on the dorsoradial side of the wrist. Ultrasonography revealed a DIT (Figure 4a) of the right wrist. Revision surgery was performed, revealing the presence of tenosynovitis associated with metallosis of the EPL, ECRB, and ECRL (Figure 4b) and intra-articular metallosis around the prosthesis. Metallosis was confirmed via histopathological examination. After the synovectomy of the radiocarpal joint, we replaced the prosthetic components with a new metal head and changed the CrCo cup to a PEEK cup (Figure 4c,d). Three months after surgery, the dorsal swelling and pain disappeared, and the ultrasound results returned to normal.

**Case 3:**

**Figure 5:**
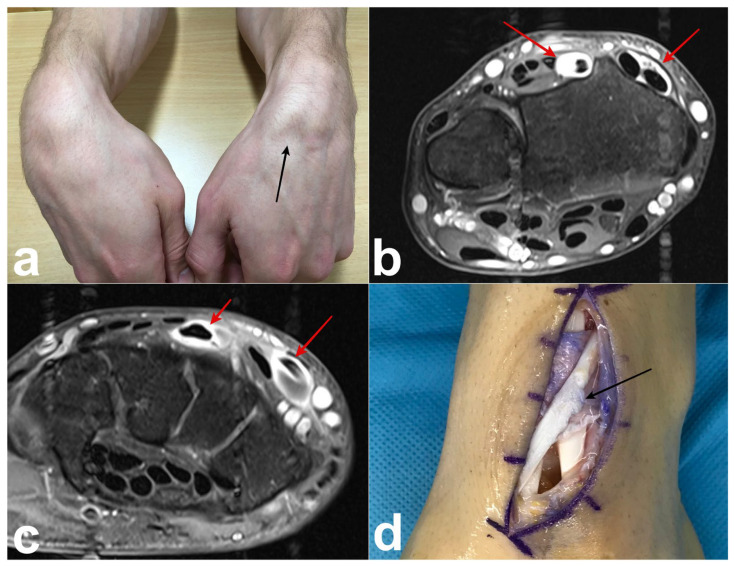
Case 3. (**a**) “heart-shaped” swelling (black arrow) of the dorso-radial aspect of the left wrist. (**b**,**c**) DIT: Tenosynovitis of the EPL, ECRB, and ECRL tendons (red arrows) of the left wrist on MRI (axial T2-weighted turbo spin-echo with fat saturation sequence). (**d**) The preoperative photograph after teno-synovectomy and the subcutaneous transposition of the EPL show evidence of tendinosis, with fraying on the superficial surface of the tendon.

A 30-year-old healthy male presented with direct trauma to the dorsal aspect of the left wrist; this was caused by changing a tire. The initial treatment consisted of immobilization and steroid infiltration in the second compartment at 8 weeks. Eight months after the trauma, we observed a “heart-shaped” swelling (Figure 5a) in the dorso-radial aspect of the wrist, painful limitations on the flexion/extension of the wrist with a range of motion (ROM) 75-0-75° (opposite 90-0-70°), a reduction in grip strength (31 kg vs. 58 kg) and pain-free active thumb mobilization. MRI showed DIT but no evidence of tendon injury (Figure 5b,c). During the surgical tenosynovectomy, synovitis of the ECRL/ECRB and EPL was observed to be continuous. Serous yellow fluid was found surrounding the tendons, and histopathological examination confirmed the presence of synovitis. The EPL tendon showed evidence of tendinosis with frailty on its superficial surface (Figure 5d). The deep aspects of the EPL tendon, as well as ECRL/ECRB tendons, showed no injury. The Lister’s tubercle of the radius was resected and the subcutaneous transposition of the EPL was performed. The patient was pain-free after four weeks of splinting and physiotherapy. Three months after surgery, the ultrasound findings returned to normal. The patient had pain-free, symmetric ROM of the wrists (90/0/75°) and grip strength (58 kg), and reported a return to full activities.

**Case 4:**

**Figure 6:**
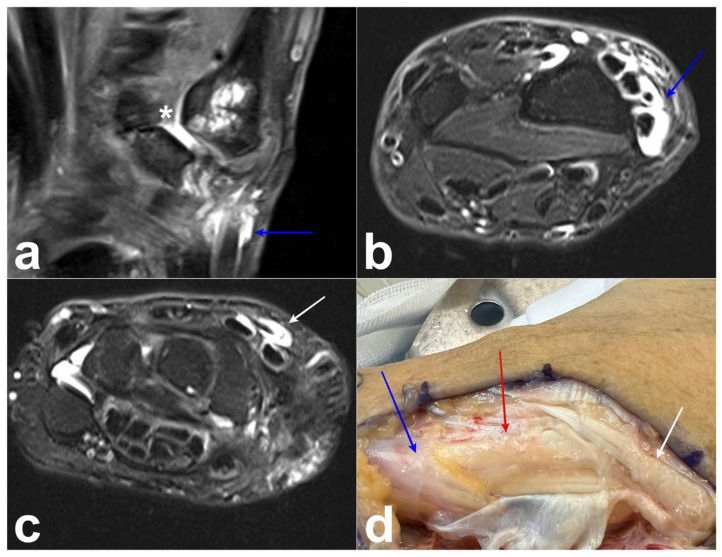
Case 4. (**a**) Conflict between the first metacarpal and scaphoid (asterisk) and tenosynovitis around the APL tendon (arrow) on MRI (coronal T2-weighted turbo spin-echo with fat saturation and dual-phase acquisition). (**b**,**c**) Tenosynovitis of the EPL, ECRB, and ECRL tendons associated with tenosynovitis of the APL and EPB tendons on MRI (axial T2-weighted turbo spin echo with Dixon fat suppression). (**d**) Preoperative photograph showing tenosynovitis of the EPL (white arrow), ECRB, ECRL (red arrow), and APL (blue arrow).

A 67-year-old woman with rheumatoid arthritis and a history of trapeziectomy 2 years before presentation presented to our clinic with swelling and pain in the dorsoradial aspect of the left wrist. MRI demonstrated the presence of a conflict between the scaphoid and the first metacarpal, and confirmed the diagnosis of DIT, as well as tenosynovitis of the abductor pollicis longus (APL) and extensor pollicis brevis (EPB) tendons (Figure 6a–c). Because of failed primary conservative treatment consisting of splinting and non-steroidal anti-inflammatory drugs (NSAID), we decided to proceed with surgery. The perioperative findings confirmed that the conflict between the first metacarpal and scaphoid caused tenosynovitis, which spread to the first, second, and third compartments (Figure 6d). The surgery consisted of the extended teno-synovectomy of every concerned tendon, joint synovectomy, interposition ligamentoplasty with the palmaris longus (PL) tendon anchovy, and the subcutaneous transposition of the EPL tendon. After six months, we noticed that the DIT had disappeared, the ultrasound was normal, and the patient was free of pain.

**Case 5:**

A right-handed 17-year-old male with a history of right distal radius fracture, treated conservatively (Figure 7a,b) a year before presentation, came to our hospital with dorso-radial wrist pain mainly after prolonged use. Clinically, we noticed tenderness over the second and third compartments, without swelling. On the radiographs, we observed malunion of the distal radius with a dorsal tilt of less than 5°. Ultrasonography and MRI confirmed the diagnosis of DIT without an anomaly on the EPL tendon. Despite immobilization and NSAID administration, the symptoms persisted. The patient underwent teno-synovectomy and subcutaneous transposition of the EPL tendon. The postoperative course was smooth, and the patient was free of pain 6 weeks postoperatively (range of motion [ROM]: F/E 55/0/80°; grip strength: Jamar 42 kg right vs. 48 kg left).

## 4. Discussion

Sixteen cases of DIT have been reported in the literature (Table 1) since 2007 [3,4,7,9,10,11,12]. The cohort consisted of eight men and eight women, ranging in age from 14 to 78 years. Among these, 15 cases were of the primary form, with no identifiable cause. One case was associated with a systemic condition [9]. A history of trauma was noted in two cases [4], while occupational or recreational predisposing factors were identified in seven cases, including activities such as playing tennis, piano, golf, video games, competitive sports, and cheerleading. Clinically, all patients presented with pain in the dorsal aspect of the wrist, often accompanied by swelling and restricted wrist mobility. The “heart-shaped” swelling reported in our description (case 3) on the dorso-radial aspect of the wrist represents the tenosynovitis affecting the tendon sheaths of the EPL and ECRL/ECRB, which diverge beyond the intersection. The term “heart-shaped” swelling is not just a descriptive choice, but also has clinical significance, as it reflects the specific pattern of fluid accumulation within the second and third compartments.

Interestingly, in most of our cases, pain was induced by wrist flexion–extension movements but was not correlated with thumb mobilization.

The diagnosis was confirmed in all cases using MRI and/or ultrasound [13,14]. The treatment details were available for 15 patients. Conservative management (including immobilization and rest) was implemented in eight cases, leading to symptom resolution in six cases. Corticosteroid injections were administered in five cases, with symptom resolution in two cases. Surgical tenosynovectomy was performed in two cases. Rupture of the EPL tendon was reported in two cases: one occurring during conservative treatment and the other following corticosteroid injection. Additionally, attrition of the ECRB tendon was observed in one case [11]. In our practice, we opted to perform surgical treatment on patients with an identifiable articular cause of DIT. In cases 3 and 5, where no tenoarticular conflict was observed, we initially attempted conservative treatment (immobilization and NSAIDs), but this proved ineffective. In our series, all patients ultimately required surgery and no complications were observed. The surgical techniques varied from one patient to another because the causes were different, and different procedures were performed concerning the intra-articular issue in cases 1, 2 and 3. However, the associated DIT consistently involved performing a tenosynovectomy along with the subcutaneous transposition of the EPL.

In most reports, the exact pathophysiological mechanism of DIT remains unclear and various hypotheses have been proposed without definitive confirmation. These include the following: (1) The vulnerability of the EPL tendon around the Lister’s tubercule due to a critical zone of watershed vascularity [4]. (2) The laceration of the ECRB due to compression stimulation by the extensor retinaculum during repeated wrist extension movements [11]. (3) Effusion in an already confined space, contributing to avascular necrosis of the tendon [7]. (4) Abnormalities of the tendons or synovial tissues related to the COMP mutation, (5) the pulley effect exerted by Lister’s tubercle over the EPL tendon, and (6) the constraining effect of the extensor retinaculum [9].

In our opinion, distal intersection syndrome is not due to friction or conflict between the tendons of the second and third compartments. We propose that inflammation starts initially either in the third compartment or in the second compartment and spreads to the other compartment through the communicating foramen in the synovial sheath of the EPL and ECRB tendons. An anatomical study of approximately 15 specimens found that this foramen measures approximatively 4–7 mm in width and 5–10 mm in length, and that it was present in all specimens. This foramen [6,7,8] could be clearly observed and documented during surgery in case 1 (Figure 2b). We suggest that due to the presence of this foramen, synovitis in one tendon can fill up the synovial sheath of the other tendon.

In cases 1, 2 and 4, we observed that different joint issues at the wrist were the direct cause of second-compartment tenosynovitis, which then spread to the third compartment. We did not observe EPL tendon lesions in these cases, and clinically, the pain was provoked by wrist motion rather than by thumb extension and retropulsion. In case 1, we found abrasion in the deep surface of the ECRB tendon.

In case 3, blunt trauma to the EPL tendon caused tenosynovitis. This is the only case in which we observed EPL tendinosis, but the lesions were superficial, in contact with the extensor retinaculum, and not in contact with ECRB/ECRL tendons. In case 5, EPL tendinitis was due to a radial fracture, which is already a well-known phenomenon (“drummer boy’s palsy”). This observation confirms our hypothesis of EPL tendinopathy and tenosynovitis due to a conflict between the Lister’s tubercle, extensor retinaculum, and EPL tendon. In these cases, inflammation probably spread from the third to the second compartment. We suggest that other causes of EPL tenosynovitis, such as compression by the thickened antebrachial fascia, osteophytes in the arthritic wrist, or a distal radius fracture [2], can lead to the same mechanism.

Zhari et al. reported a clinical case of a 60-year-old male with dual intersection syndrome, indicating a proximal intersection syndrome concomitant with a distal intersection syndrome. It is not specified which therapy or evolution the patient went through [12]. In case 4, the same phenomenon was observed. Inflammation clearly began in the scapho-metacarpal joint and spread to the 1st, 2nd and 3rd compartments.

One limitation of this study is the short-term follow-up, which prevents us from mentioning the long-term outcomes or recurrence rates of DIT in the five cases. We also only based our results on clinical exams and ultrasound in four cases, but did not repeat the MRI examination. A prospective study with a larger patient cohort to determine the optimal conservative therapy [15,16] or timing for surgery would be of great interest. However, several questions remain unanswered. First, why is DIT rare if cadaveric studies show that foramina are present in all specimens [6]? If this communication is universal, EPL or ECRB tendinitis should invariably lead to DIS. Could its apparent rarity be attributed to underdiagnosis due to a limited awareness of the condition and are there still unidentified pathomechanical factors at play? Second, the optimal treatment approach remains unclear. Given the rarity of this condition, conducting controlled studies is challenging. While steroid injections are commonly used, they may compromise the vascularity of the EPL tendon, potentially increasing the risk of rupture. In addition, prolonged tenosynovitis contributes to ischemia and tendon rupture. In our cases, immobilization and NSAIDs were ineffective. This raises a critical question: should surgical intervention be considered earlier in the treatment course, particularly when no clear intra-articular cause is identified?

## 5. Conclusions

These cases highlight the specific underlying conditions that may contribute to the development of DIT and provide new insights into its pathophysiology. Our observations suggest that wrist joint or bone disorders may play a significant role in its occurrence, which has not yet been described in the literature. Lesions in different tendons suggest the involvement of distinct pathological mechanisms. Further studies on this condition could provide deeper insights into its pathomechanics and help refine the treatment strategies.

## Figures and Tables

**Figure 7:**
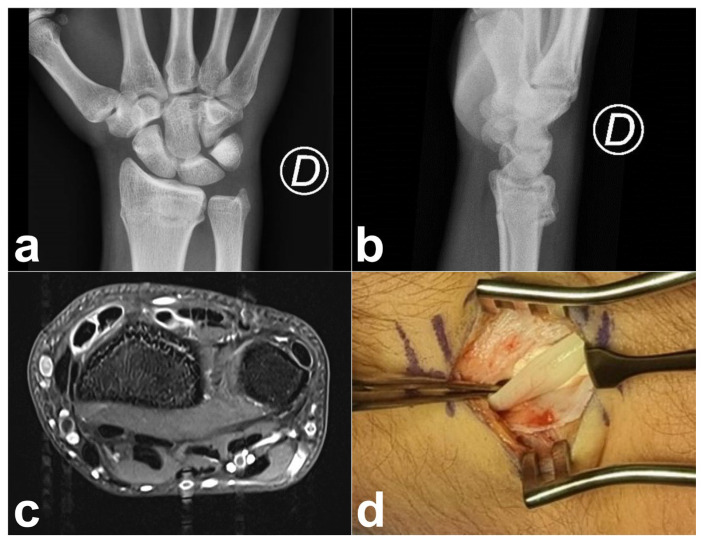
Case 5. (**a**,**b**) Preoperative radiograph showing a distal radial fracture with minimal dorsal tilt. (**c**) DIT: Tenosynovitis of the EPL, ECRB, and ECRL tendons of the right wrist on MRI (axial T1-weighted turbo spin-echo with fat saturation and Dixon-based reconstruction). (**d**) Perioperative photograph after teno-synovectomy and subcutaneous transposition of the EPL tendon.

**Table 1:** Articles on distal intersection tenosynovitis. CSI, corticosteroid injection. US: ultrasound. MRI: magnetic resonance imaging.

Article	Patients, Sex, Age (Years Old)	Predisposing Factors	Clinical Presentation	Imaging, Findings	Therapy	Outcomes
Alter [3]	A. Male, 29 B. Female, 14 C. Male, 17	A. Playing video games B. Competitive cheerleading C. Tennis	Dorsoradial pain, paresthesia and edema	US and MRI	A. Conservative B. Conservative C. Failed conservative treatment motivating a teno-synovectomy	A–B. Successful C. Successful
Parellada [4]	3 females 2 males (mean 49)	Trauma in 2 cases	Pain on the dorsal and radial aspect of the wrist	MRI	Conservative management in 4 cases Surgical teno-synovectomy in 1 case	Successful
Mattox [7]	Female, 38	Not specified	Painful swelling	US	Conservative	EPL tendon rupture
Ideta [9]	Female, 40	COMP mutation	Tenderness. Wrist stiffness	MRI	Surgery: teno-synovectomy	Successful
Sunagawa [11]	A. Male, 20 B. Male, 23	tennis player	Swelling and tenderness. Painful wrist extension.	US MRI (showing partial laceration of the ECRB)	A. CSI B. CSI and splint failed motivating a surgical teno-synovectomy	A. EPL rupture B. Successful
Nam [10]	A. Female, 38 B. Female, 41 C. Male, 61	A. Housework B. Piano C. Golf	Pain, stiffness, edema	MRI and US	A. Conservative and CSI B. CSI C. CSI	A–C. Successful B. Improvement
Zhari [12]	Male, 60	Not specified	Pain and swelling, local crepitation	MRI and US	Not specified	Not specified

## Data Availability

All data generated or analyzed during this study are included in this article. Further inquiries can be directed to the corresponding author.
